# Arm Propulsion in Front Crawl Stroke

**DOI:** 10.3390/sports13010006

**Published:** 2025-01-02

**Authors:** Cristian Romagnoli, Vincenzo Bonaiuto, Giorgio Gatta

**Affiliations:** 1 Department of Human Science and Promotion of Quality of Life, San Raffaele Open University, 00166 Rome, Italy; 2 Sport Engineering Lab, Department of Industrial Engineering, University of Rome Tor Vergata, 00133 Rome, Italy; vincenzo.bonaiuto@uniroma2.it; 3 Department for Life Quality Studies, University of Bologna, 40100 Bologna, Italy; giorgio.gatta@unibo.it

**Keywords:** power balance, propulsive force, drag power, propulsive power

## Abstract

**Objectives**: This study aims to determine the propulsive force and effective arm area contributed by the propulsion through the dynamic balance (power balance) between drag and propulsive power in swimming crawl performance. **Methods**: Ten male swimmers participated in the study. The athletes conducted the crawl trials at a constant velocity using only the upper limbs. Data were collected using a Spectro instrument to measure the drag and 3D video analysis for kinematic of upper limbs movement. **Results**: The power balance was confirmed through the Bland–Altman estimation (estimated bias 8.5) and was also demonstrated by a one-way analysis of variance that does not show statistical differences. Subsequently, by applying the power balance, the effective propulsive area could be estimated. The result shows an increase of ~8.5% over the value at the hand area used to verify the power balance. This value appears to be attributable to a percentage of the forearm area to propulsive action. **Conclusions**: This information will allow athletes and coaches to constantly monitor the propulsive force and power, providing useful data on arm movement and swimming technique. Indeed, deeper knowledge about the athlete’s swimming technique can reduce the possibility of suffering micro-traumas in the elbows and shoulders.

## 1. Introduction

In all forms of locomotion (such as walking, cycling, kayaking, and swimming), there is a relationship between propulsive power and drag (aerodynamic or hydrodynamic) that limits the movement of each subject’s performance. In crawl swimming, the forward movement is determined by the upper limbs’ alternating actions and the lower limb’s kick pattern (two-, four-, or six-beat kick) contribution [1]. For this reason, in this condition, it is fundamental to improve the propulsive force and relative power or reduce the drag.

As highlighted in the literature, 87–90% of crawl swimming propulsion is generated by the action of the upper limbs [2,3,4,5]. Such studies suggest that the muscle activations, measured through the sEMG, as the flexor carpi ulnaris, biceps brachii, latissimus dorsi, and triceps brachii are highly activated during the underwater phase and are critical for maintaining swimming speed during races [6,7,8,9].

According to Toussaint [10], in competitive swimming, this percentage can be divided into 61% of work per stroke to overcome drag and 39% being converted into the kinetic energy of water during push-off. On the other hand, leg kicking seems to only partially contribute to the overall swimming speed at approximately 10–13%, because it acts as a stabilizer during the swim [5].

Counsilman’s theories [11] have played a fundamental role in the study of propulsion in swimming because they demonstrated the presence of particular curvilinear trajectories of the hand during the phases of the stroke in the water. Based on these theories, many studies [12,13,14,15,16,17,18,19] have highlighted that these movements have a key role in generating propulsive force by exploiting the hand’s drag and lift forces.

Therefore, the propulsive force (F_p_), defined as the component of the force in the swimming direction, is equal to the sum of the projections on such axes of the drag and lift forces generated by the hand [20,21]. Thus, we can consider the swimmer’s advancement in the water as the combined contribution of lift and drag force related to the upper limb (i.e., hand or hand–forearm compartment) during the stroke [22]. The F_p_ can be determined by the following hydrodynamic equation as suggested by Alexander and Goldspink [23] and Toussaint et al. [24]:

(1) $$F_{p}=\frac{1}{2}\rho C_{dh}A_{h}v_{h}^{2}$$

where ρ is the density of the water [kg m^−3^], A_h_ is the hand area [m^2^], *v*_h_ is the hand velocity [m s^−1^], and C_dh_ is the hand drag coefficient.

In contrast to the propulsive forces, the drag of the water (D) acts on the swimmer’s body, slowing them. The drag force D can be calculated as follows:

(2) $$D=K_{a}v_{sw}^{2}$$

K_a_ is the active drag coefficient estimated from passive drag K_p_ multiplied by 1.5, as suggested by [25,26], and v_sw_ is the swimmer velocity.

In a front crawl swimming simulation where the athlete uses only the upper limbs (as in our work), the power produced by the arms (W_p_) will be equal to the F_p_ exerted by the athlete through the movement of the arms. So, the propulsion power can be estimated as follows:

(3) $$W_{p}=F_{p}{(v}_{h})$$

where v_h_ is the velocity of the hand. In contrast, the power drag (W_D_) can be estimated as:

(4) $$W_{D}=D{(v}_{sw})$$

where v_sw_ represents the swimmer’s speed.

When the swimmer reaches a constant pace, the balance between the propulsive power (W_p_) and the drag power (W_D_) occurs [10].

(5) $$W_{p}=W_{D}$$

The power balance hypothesis in swimming and kayak performance has already been introduced theoretically by Kolmogorov and Duplischeva [27], Schultz and Webb [28], and Touissaint and Beek [10] and demonstrated by Gatta et al. [29] and Romagnoli et al. [30] through tethered swimming and video analysis 2D integrated with the e-kayak system [31], respectively.

To verify the power balance, it is fundamental to precisely estimate all the variables reported in Equation (1) and, in particular, the value of the area effectively involved in the propulsion ($A_{h}^{*}$). Several studies have investigated the effects related to the shape of the hand [32,33,34] or investigated the fingers’ positions [35,36] to evaluate the useful surface area used in the propulsion assessment. Indeed, the estimation of this surface is widely discussed in the literature. Some authors propose using only hand surfaces, while others include part of the forearm in the analysis [20,22,37,38].

From a comparison of these studies, it seems that the Schleihauf method [22,39] on the upper limb reconstruction is accurate, stable, less sensitive to random errors in single points, and provides very reliable results when used in the laboratory. Contrarily, the Berger method [20], which requires a limited number of points for the 3D swimmer’s upper limb movement reconstruction, could be more affordable in tests performed in specific conditions and, in particular, when some of the points could be hidden, for example, by the turbulence of the water during the stroke.

For this purpose, several estimation methods to retrieve the value of this parameter are available in the literature [35,40,41], and what emerges from their analysis is that the results strongly depend on the quality of the input images and the suitability of the positions of the markers for the reconstruction of the upper limb movements. Furthermore, the definition of the hand surface that is useful for propulsion also depends on the hand orientation angle and this the can change, influencing the useful area during underwater movement [41]. During these propulsive phases, the propulsion results from the combination of lift and drag. It is possible to determine an *angle of attack*, which can be defined as the angle between the hand’s line of motion and the hand’s plane; this was observed to be between 20 and 70 degrees [20,39,42,43]. From these analyses, the authors believe that for a proper estimation of an optimal propulsive force value, it is valid and indicative to consider the coefficient of drag (C_dh_) as having a mean value of approximately 1.2, as suggested by Bilinauskaite et al. [44] and Bixler and Riewald [37].

Since the beginning of the 20th century, many authors have tried to measure the forces during swimming using different methods. Tethered swimming was the first used by Houssay [45], Cureton [46], Karpovich, and Pestrecov [47]. In another way, Lilejstrand and Stenström [48] and Di Prampero et al. [49] led studies on propulsive forces and drag from energy consumption. Different authors have improved these two methods [25,50,51,52,53,54,55,56]. More recently, pressure transducers or strain gauges were fixed on the palmar face of the hand to evaluate the propulsive forces of the hand [57,58,59]. All these studies presented only descriptive results without any question on the relationships between the force production and the swimmer’s speed [60]. Only Berger et al. [61] compared the MAD system [10] with 3D video analysis, confirming that calculating the propulsive forces from a three-dimensional kinematic analysis, combined with lift and drag coefficients, provided realistic values for the mean propulsive force during front crawl swimming.

Based on previous considerations, when the swimmers move at a constant pace, the power balance is verified as follows:

(6) $$K_{a}v_{sw}^{3}=\frac{1}{2}\;\rho \;C_{dh}\;A_{h}\;v_{h}^{3}$$

When using the method proposed by Gatta et al. [26] and Romagnoli et al. [30].

According to this hypothesis, this study aims to define the effective propulsive area, $A_{h}^{*}$. The power balance will be used to theoretically estimate the percentage of forearm area in addition to the hand area, which has to be taken into account (if the value of C_dh_ = 1.2 is used).

## 2. Materials and Methods

### 2.1. Subjects

Ten sub-elite male swimmers served as the participants (age: 24 ± 3 years, height: 1.79 ± 0.90 m, mass: 76.73 ± 10.29 kg). Their best performance in the 100 m front crawl in a 50 m swimming pool was 56.45 ± 3.96 s. Each athlete held competitions at the national level and had a training load of no less than 12 h per week. Written informed consent was obtained, the procedures followed the Helsinki Declaration of 1975, and approval was obtained from the Internal Research Board of “Tor Vergata” University of Rome for this study. The tests were performed in a 25 m × 12.5 m indoor pool (water temperature 28 °C).

### 2.2. Procedures

#### 2.2.1. Drag Measurement

The swimmer’s drag at different velocities was measured following the methods proposed by Gatta et al. [26] and Cortesi et al. [25]. Each participant was connected to the Spectro instrument (Swim-Spektro, Talamonti Spa, Ascoli Piceno, Italy) through a steel wire and towed at five velocities (1.0; 1.3; 1.6; 1.9; 2.2 m/s). Towing force was calibrated by pulling constant loads (26, 40, 68 N, load Spektro) at a constant speed (1.0 m × s^−1^) [25].

Participants performed the passive tow test using their best horizontal hydrodynamic position [25]: the arms extended forward and close to the head, maintaining the hands together. In addition, the lower limbs were kept at their maximum extension. The average values of the drag force (F_d_) were measured between 10 and 20 m from the starting wall. The best test, with the lowest F_d_ values, was selected for the present analysis.

The data are reported graphically to allow us to retrieve the value of the passive drag coefficient (K_p_), which was corrected in the active drag coefficient (K_a_), as proposed by Gatta et al. [26], by multiplying it by the crawl swimming factor (equal to 1.5). Finally, the K_a_ value computed for each subject was applied in the different tests, obtaining a measure of the drag force F_d_ and the relative drag power W_d_.

#### 2.2.2. The 15 m Swimming Test

Before conducting the 15 m swimming test, each athlete performed a specific warm-up on dry land and in water: 5’ of static stretching and 5’ of dynamic stretching for the upper limbs, followed by eight trials of 25 m front crawl at increasing velocity, between each trial 2–3′ of recovery.

The 15 m swimming tests (at a controlled constant velocity) were performed in crawl style, eliminating the action of the legs, which were held in place using a lace and supported by a small pull-buoy. In this way, propulsion is developed only by the action of the upper limbs, avoiding the legs, which can introduce a further propulsive increase that is not easy to estimate.

The estimation of the propulsive surface of the hand was considered using a planimetric measurement with closed fingers. The average hand areas (0.0165 ± 0.001 m^2^) were computed for all the swimmers using a specific software program (Universal Desktop Ruler, v3.3.3268, AVPSoft, USA). The test/retest ICCs of the area of the hand were 0.99.

#### 2.2.3. Simi Motion Analysis

In the quasi-static approach, the hand’s velocity significantly influences the size of the hand and forearm propulsive forces. The swimmer’s hand velocity on the x-axis was measured using 3D stereophotogrammetry frame by frame [62,63,64,65]. An investigation of the accuracy of three-dimensional space reconstruction using the direct linear transformation technique (3D-DLT) [66] was used to calibrate the control object (x = 1.5 m; y = 45 cm; z = 49 cm) through 8 markers (4 for each side). Two underwater cameras were synchronized (GoPro, Hero 4, Woodman Labs, Inc., San Mateo, CA, USA) through the Remote GoPro control; they were set at 120 fps, as suggested by Cronin et al. [63], and were positioned in front and to the right side of the swimmer’s movement direction. During each test, the swimmer was recorded for the last 15 m using another camera (Huawei mate 20lite—FHD 1080p 1920 × 1080 resolution) to monitor the stability of the average velocity.

The subjects were asked to perform different tests at progressively increased mean velocity: slow (1.00 to 1.20 m/s), medium (1.21 to 1.50 m/s), and fast (1.51 to 1.75 m/s). The right hand’s marker (wrist) was tracked using SIMI motion software (SIMI^®^ Really Motion System GmbH, version 8.0, Munich, Germany) through stereophotogrammetry (frame by frame), simultaneously for lateral and frontal view. The 3D average velocities (ABS) were estimated during the propulsive phase because this was demonstrated to have a good agreement and accuracy in all directions compared with the elbow and shoulder, as the trajectory of the hand is commonly used to identify and characterize the stroke phases [62].

The manual digitizing was carried out by two experienced operators (>1000 h of digitizing) who used anatomical criteria alongside the software’s features (e.g., zoom function) to identify the wrist coordinates using the 3D Simi motion analysis software. This methodological approach has previously demonstrated high reliability in competition-based athletic settings [62,63,64,65,67].

To evaluate the inter-operator repeatability of the measures, each operator, for each swimmer’s velocity, was required to manually digitize the hand’s coordinates on each video frame acquired by both cameras. Furthermore, each operator repeated the video analysis five times to ensure intra-operator variability. The repeatability results showed good accuracy in retrieving the hand’s coordinates between the two operators (ICC = 0.92) and among repeated analyses by the same operator (ICC = 0.93) [63].

#### 2.2.4. Variables Calculated for Each Athlete

To verify the power balance, the hand propulsive force (F_p_) and power produced by the action of the upper limbs were calculated, and the Newtonian Equations [23] were used. From the Equations (5) and (6), the value of C_dh_ was set to 1.2, as suggested by [44,68].

Based on the theory of power balance, it is possible to estimate the effective propulsive area ($A_{h}^{*}$) (i.e., the areas of the hand and forearm together in m^2^) through the following equation:

(7) $$A_{h}^{*}=\frac{W_{D}}{\frac{1}{2}\rho v_{h}^{3}}$$

But if it is believed that only the A_h_ is propulsive, it is possible to estimate the new coefficient for hand (C_dhe_) as:

(8) $$C_{dhe}=\frac{W_{D}}{1/2\rho A_{h}v_{h}^{3}}$$

### 2.3. Statistical Analysis

Unless otherwise specified, the results are presented as mean and standard deviation (M ± SD). The Kolmogorov–Smirnov test was used to validate the assumption of normality. One-way analysis of variance (ANOVA) was used to detect significant differences between W_D_ and W_p_. Bland and Altman plots [69] were used to analyze the level of agreement between W_p_ and W_D_. In addition, Lin’s concordance correlation coefficient (CCC) [70] was used to evaluate the degree to which pairs of observations fall on the 45° line through the origin. Furthermore, to describe the values of the concordance correlation coefficient, the scale proposed by Mcbride [71] was used.

In addition, the coefficient of repeatability (RC) and the effect sizes (ES) were also calculated according to [72], and Cohen’s d was used to define the intra-observer difference between two trials for each range’s velocity [73], where the small effect was 0.1, the moderate was 0.3, and the large was 0.5 [74].

## 3. Results

The descriptive results of kinematic and dynamic variables are presented in Table 1 with M ± SD. Table 1 reports the average values of the tests at different velocities described above.

**Figure 1:**
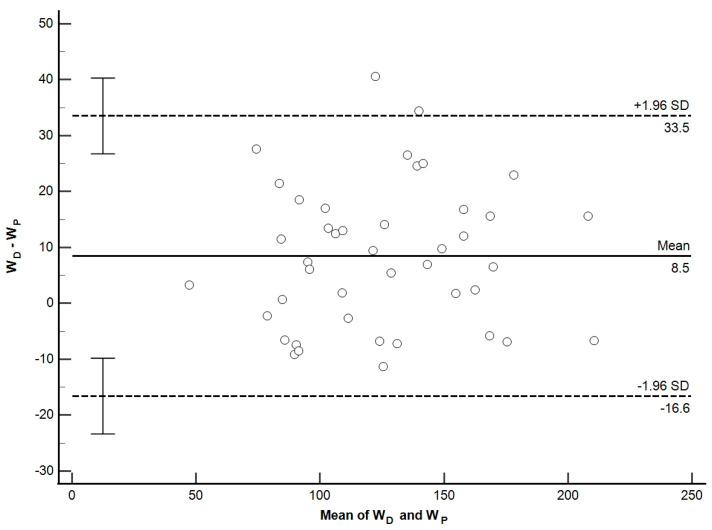
Bland–Altman plot displays a scatter diagram of the differences plotted against the averages of the two measurements WD and WP. Horizontal lines are drawn at the mean difference (normal line), and at the limits of agreement (dashed line). The 95% limits of agreement (LoA) are defined as the mean difference ± 1.96 SD.

Bland and Altman’s plot shows that the W_p_ values agree with W_D_ (Figure 1).

The estimated bias for the power is 8.50 W (95% CI 4.5450 to 12.4132), and the upper and lower limits of agreement are 33.50 (95% CI 26.7583 to 40.3102) and −16.60 W (95% CI 23.3520 to −9.8001). In addition, the coefficient of repeatability has been calculated as suggested in [72], RC = 29.82 (95% CI 24.6396 to 37.7852).

**Figure 2:**
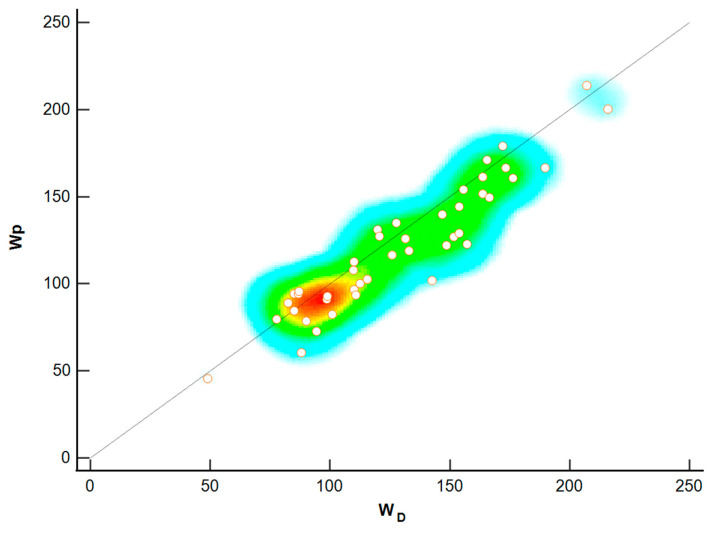
Lins’s concordance correlation coefficient (CCC), where the dots represent the correlation between W_D_ and W_P_. The different colors represent respectively the density distribution of values (e.g, high density = red).

Lin’s concordance correlation coefficient (CCC) shows a high strength of agreement (Figure 2) between W_D_ and W_p_, with a concordance correlation coefficient = 0.95, 95% CI = 0.87 to 0.95, Pearson correlation (*p*) = 0.94, and bias correction factor (C_b_) = 0.97.

Table 2 reports the one-way ANOVA analysis and the effect size (ES). The results do not show significant differences between the two considered methods (*p* < 0.05).

## 4. Discussion and Implications

In the several studies on swimming propulsion referred to in this paper, very different values are reported, which can hardly be discussed because they depend on the characteristics of each swimmer and the relative speed that they produce. This study aims to verify the *power balance theory* by calculating, through the three-dimensional kinematic analysis, the W_p_ and F_p_ developed by the athlete’s hand during the crawl at a constant pace to cover the W_d_. From the comparison between W_D_ and W_p,_ the one-way ANOVA confirms no significant difference between power values (*p* = 0.298) (Table 2). In addition, the differences observed in the Bland–Altman plots (Figure 1) are small (bias:8.50 with limits of agreement between 33.50 and −16.60). In addition, Lin’s concordance correlation coefficient (Figure 2) confirms the strengthened agreement between the method proposed, with CCC = 0.91 and Pearson coefficient = 0.94, representing the measure of how far each observation deviates from the best-fit line.

These results confirm that the power balance theory is verified when the swimmer moves at a constant pace velocity [29,30]. For this reason, thanks to the *power balance theory*, estimating the effective area with the inverse formula is possible using the W_p_.

The issue surrounding the effective propulsive area of a swimmer’s limb, and consequently the propulsive force generated, is a well-researched topic. Initial studies, such as those by Counsilman [11] (1971) and Schleihauf [16,22], examined hand models to estimate the propulsive force (F_p_) using relatively unspecific methods, focusing primarily on drag and lift coefficients [33,36,40,75,76]. Other researchers have expanded on this by including both the hand and forearm areas as effective propulsive areas when estimating F_p_, employing more sophisticated calculation techniques to determine drag coefficients for the hand (1.15–1.27) and forearm (0.65) [20,37,44,77,78,79].

Today, this research supports using a drag coefficient (C_dh_) equal to 1.2 to calculate the propulsive force based on the hand area alone. Following power balance theory, the effective propulsive area (A_eff_), combining the hand and forearm areas (in m^2^), can be estimated using Equation (7). The comparison between the measured (*A_h_*) and the estimated ($A_{h}^{*}$) values shows a difference of approximately 8.5% (Table 1). These findings indicate that, when calculating propulsive power (W_p_) using the F_p_ estimated using Equation (1), adding about 8.5% of the forearm area to the hand area is necessary while maintaining a drag coefficient of C_dh_ = 1.2.

If, on the other hand, we consider propulsion to be exclusively related to the hand area to estimate values of W_p_ or W_d_, we would have to know the correct value of C_dh_. Carrying out the inverse formula (Equation (8)), the average C_dhe_ to be considered in this situation is 1.3 ± 0.15, slightly higher than that indicated in the literature (C_dh_ = 1.2) [37,44]. Based on these indications, to estimate the W_p_ or W_d_ correctly_,_ it is possible to use two different methods:

- Consider the A_eff_ = A_h_ + (~ 8.5%) with C_dh_ = 1.2.
- Consider only the hand’s area and use a value of C_dhe_ of about ~1.3.

There are limitations in the interpretation of the results of this study. The restricted number of athletes involved in the study could have affected the significant differences between the two considered methods. Furthermore, the 3D analysis space taken into account for the measurement estimation focused only on the right view of the swimmer. Additional studies could be conducted using the same proposed methodology and performed with elite male and female athletes.

## 5. Conclusions

This pilot study used 3D video analysis integrated with Spectro device and a theoretical approach to determine the propulsive power, drag power, and the upper limb’s effective propulsive area during crawl (only upper limbs) at a constant pace velocity. Despite the two proposed methods of estimating W_P_ and W_D_, namely varying the C_dh_ or considering the area of the hand plus an ~8.5% that depends on the location of the forearm, the results of this study show no significant differences between W_p_ and W_D_, even considering only the hand area and C_dh_ = 1.2 for estimating W_p_. This information may be useful for athletes and coaches to properly monitor, train, and assess the propulsive force and power generated by the athletes during the stroke and when they use a different swimming paddle size during training. In addition, monitoring these variables not only provides useful data on arm movement and swimming technique but, through the optimization of these aspects, it is possible to reduce the risk of injuries [80] (e.g., micro-traumas in the elbows and shoulders) related to overload or improper movements and promoting, in this way, the athlete’s overall fitness and wellness.

## Figures and Tables

**Table 1:** Kinematic and dynamic variables related to the swimmers: K_p_ (coefficient of passive drag), K_a_ (coefficient of active drag), *A_h_* (hand area), $A_{h}^{*}$ (estimated area relative hand + forearm), v_sw_ (swim velocity), v_h_ (hand velocity), W_D_ (drag power), W_p_ (propulsive power), F_p_ (propulsive force), and C_dhe_ (hand drag coefficient estimated).

Coefficient of Passive Drag [K_p_]	Coefficient of Active Drag [K_a_]	A_h_ [m^2^]	$A_{h}^{*}$ [m^2^]	Swim Velocity (v_swm_) [m/s]	Hand Velocity (v_h_) [m/s]	W_D_ [W]	W_p_ [W]	F_p_ [N]	C_dhe_
28.16 ± 2.34	42.23 ± 3.52	0.0164 ± 0.001	0.0178 ± 0.003	1.44 ± 0.15	2.30 ± 0.25	129.20 ± 37.91	120.71 ± 37.12	51.72 ± 10.56	1.30 ± 0.15

**Table 2:** One-way ANOVA (post hoc test: Tukey–Kramer—significance was set at *p* < 0.05) between W_D_ and W_p_.

W_D_ [W]	W_p_ [W]	*p*	ES	95% CI [ES]
129.20 ± 37.91	120.71 ± 37.12	0.298	0.22	0.12 to 0.34

## Data Availability

The data that support the findings of this study are available from the corresponding author upon reasonable request.
